# Evaluation of the etiology of persistent iritis after cataract surgery

**DOI:** 10.1186/s12348-019-0170-2

**Published:** 2019-02-18

**Authors:** Kristin Neatrour, Allison McAlpine, Timothy Brooks Owens, Rupal H. Trivedi, Lynn J. Poole Perry

**Affiliations:** Storm Eye Institute, Medical University of South Carolina, 167 Ashley Avenue, Charleston, SC 29425 USA

**Keywords:** Post-operative uveitis, Post-operative management, Iritis, Inflammation, Risk factors, Cataract surgery complications, Pseudophakia, Epidemiology, Incidence

## Abstract

**Background:**

The purpose of this study was to evaluate patients with persistent iritis after cataract surgery to determine its incidence and risk factors. Adjusting the management of patients at risk could allow for a more predictable post-operative course and outcome. A retrospective chart review was performed of patients who had post-operative iritis longer than 1 month after cataract surgery during a 2-year period at Storm Eye Institute at the Medical University of South Carolina (MUSC) in Charleston, South Carolina. Patient demographics and various pre-operative, intra-operative, and post-operative factors were analyzed for trends.

**Results:**

Thirty-nine patients (49 eyes) met the inclusion criteria, and this group was compared to a control cohort of 40 patients (66 eyes) who did not have persistent iritis after cataract surgery. The overall incidence of post-operative iritis was 1.75%. In all patients with post-operative iritis lasting greater than 1 month, African American race and pupil expansion device use were statistically significant factors. After excluding patients with a history of ocular inflammation or known inflammatory or autoimmune diagnosis (1.20% incidence), there were still a significantly higher proportion of African Americans compared to the control group. When patients with post-operative iritis of less than 6 months in duration were additionally excluded, the incidence was 0.32%, and history of diabetes was statistically significant in addition to race.

**Conclusions:**

Risk factors for persistent iritis after cataract surgery include being diabetic, of African American racial background, and pupil expansion device use. These patients can be better informed of the higher risk of prolonged inflammation in their post-operative course, and peri-operative management can be tailored accordingly.

## Background

Cataract surgery techniques have evolved significantly in recent years. Modern surgical advances and medications have led to improved peri-operative management of inflammation and better outcomes, even with challenging uveitic eyes [1]. Routine cataract surgery results in the release of inflammatory mediators, leading to some degree of post-operative inflammation in all patients. This post-operative inflammation is usually easily controlled with topical steroid tapers and typically resolves by 1 month after surgery.

In the presence of certain risk factors, such as a history of uveitis, this post-operative inflammatory period may be prolonged for weeks or months, punctuated by periods of “rebound” inflammation when topical steroid drops are tapered. Studies have shown that the recurrence rate of uveitis following cataract surgery is as high as 51% [2]. A study performed at Vanderbilt University found a significant association between postsurgical uveitis and intra-operative complications, as well as worse visual acuity outcomes. The median duration of inflammation was 10 months [3]. In a review of rheumatoid arthritis patients undergoing cataract surgery, elevated pre-operative rheumatoid factor (RF) serum titers were associated with 1+ aqueous cell 1 month after surgery. Interestingly, analysis of patients with this level of post-operative cell and low pre-operative RF titers revealed that 75% of these patients had diabetes [4].

In the absence of a known predisposing etiology such as a history of uveitis or surgical complications, however, some patients still experience a prolonged course of inflammation after cataract surgery. The unique characteristics shared by this subset of patients have not been previously studied.

The purpose of this study was to evaluate patients who developed prolonged post-operative iritis, but who had no known risk factors, in order to determine the incidence and possible underlying etiologies or predisposing factors. To investigate this, a 2-year retrospective chart review was performed of patients at Storm Eye Institute at the Medical University of South Carolina (MUSC) who developed post-operative iritis persisting longer than 1 month after cataract surgery. Patient demographics and various pre-operative, intra-operative, and post-operative factors were evaluated for trends. These results were then compared to a cohort of patients who did not have persistent post-operative iritis to evaluate for statistically unique characteristics.

## Methods

The MUSC Institutional Review Board approved this retrospective study, and the research followed the tenets of the Declaration of Helsinki. The data requests were submitted through the Services, Pricing, and Application for Research Centers (SPARC) at MUSC, and this process complied with the Health Insurance Portability and Accountability Act. A list of medical record numbers was provided for all patients in the 2-year time period (from November 2013 to September 2015) whose charts had International Classification of Diseases (ICD) codes for both “pseudophakia” (ICD-10 Z96.1) and “iridocyclitis” (ICD-10 H20–H20.9). These charts were then analyzed for pre-operative, intra-operative, and post-operative characteristics as discussed below. To determine the incidence of persistent post-operative iritis, another SPARC data query produced the number of total procedures performed in the 2-year time period that were billed with a Current Procedural Terminology (CPT) code of 66982 or 66984. The incidence was calculated strictly based on surgery date occurring within the study period, whereas the subsequent analyses included the cohort of patients with clinic visits for prolonged post-operative iritis in that time period in order to study a larger sample size.

Pre-operative characteristics included basic demographics of age, gender, and race. It was noted if this was the first or second eye undergoing cataract surgery, as well as the course of the other eye, if applicable. If patients had a history of ocular inflammation, then the ophthalmic diagnosis, underlying systemic inflammatory diagnosis with supporting lab or imaging studies, baseline systemic or topical anti-inflammatory medications, and pre-operative anti-inflammatory prophylaxis regimen were recorded. No prophylaxis was prescribed unless there was a uveitic history, in which we routinely prescribe an oral prednisone taper at the time of surgery. All ophthalmic diagnoses, prior ocular surgeries, prior ocular trauma, and other medical diagnoses were also included. For patients with glaucoma, medical and surgical treatments were listed. Diagnosis of diabetes was reported along with duration, peri-operative hemoglobin A_1c_ (HbA_1c_), status of diabetic retinopathy (DR) including macular edema, and if a recent intravitreal injection was administered, based on information available in the chart. The type of cataract (senile, uveitic, or traumatic) and grade (for nuclear, cortical, and posterior subcapsular classifications) were noted.

Intra-operatively, the attending surgeon’s name was documented in the operative note, but the resident’s level of participation in the case was typically not included. There were five attending cataract surgeons at MUSC during this time period. It was noted if femtosecond laser-assisted cataract surgery (FLACS) was performed and if intracameral antibiotics were administered, if known. Any documented findings outside of the standard operative note template were recorded, including the use of a pupil expansion device, intra-operative floppy iris syndrome (IFIS), posterior capsule violation, anterior vitrectomy, and intraocular lens (IOL) suture fixation and the use of triamcinolone, and if the cataract surgery was combined with any other procedures, such as a trabeculectomy or minimally invasive glaucoma surgery (MIGS). The type of IOL (e.g., SN60WF) and implanted location (posterior chamber, sulcus, or anterior chamber) were also recorded.

Post-operative topical drops used at our institution include a month of a topical non-steroidal anti-inflammatory drug (typically ketorolac four times daily) and a 4-week taper of prednisolone (four times per day for 1 week followed by a weekly tapering off the drop). Post-operative findings included the duration of persistent iritis (in months) as well as the extent of anterior chamber cell and flare (evaluated with slit lamp examination according to the Standardization of Uveitis Nomenclature system) [5]. Patient symptomatology was noted if specified in the record. Response to topical therapy was categorized as rapid or indolent and persistent, and any topical and/or systemic adjunctive treatment was listed. Any documentation indicating good or poor compliance was included. Lastly, post-operative best-corrected visual acuity (BCVA) and maximum intraocular pressure (IOP) were recorded.

For the control group, a similar SPARC data request was submitted for a list of all patients in the same time period whose charts had ICD codes for pseudophakia but not iritis. A random integer generator was used to select 40 patients (66 eyes) for the control group. The same pre-operative and intra-operative characteristics were recorded, as well as the post-operative BCVA and maximum IOP.

Patient and peri-operative characteristics were analyzed with descriptive statistic calculations. Continuous variables were analyzed with a two-tailed *t* test and categorical variables were evaluated with the Fisher exact test. Visual acuity based on the Snellen chart was converted into logarithm of the minimum angle of resolution (logMAR) units. A *p* value of < 0.05 was considered statistically significant for the analysis.

## Results

During the 2-year time period, 2169 cataract surgeries were performed at MUSC. Of these cases, 38 eyes had post-operative iritis lasting longer than 1 month (1.75%). Eliminating eyes with a prior history of ocular inflammation or pre-existing systemic inflammatory or autoimmune diagnosis led to an incidence of 1.20% (26 eyes). After excluding the eyes with both post-operative iritis less than 6 months in duration and a prior history of inflammation, the incidence was 0.32% (7 eyes).

**Fig. 1:**
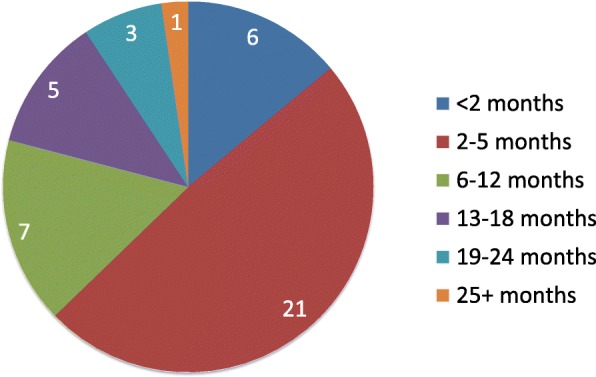
Number of eyes versus the length of iritis. Legend: Fig. 1 shows the distribution of patients with prolonged inflammation based on the duration of iritis in months

Thirty-nine patients (49 eyes) were seen in the clinic for the evaluation and management of prolonged post-operative iritis during the study time period. Figure 1 shows the relative distribution of patients categorized by the duration of post-operative iritis. Six [6] patients were excluded from this subset analysis because 3 patients were lost to follow-up and 3 patients had ongoing iritis at the time of data collection (*n* = 43). The average duration in this subset was 7.2 (± 7.7) months, and the median was 4 months.

The analysis of all patients comparing the cases of prolonged post-operative inflammation with the control cases showed that African American race and the use of an intra-operative pupil expansion device were statistically significant. When patients with prior ocular inflammation or pre-existing diagnosis were excluded, African American race remained statistically significant. After additionally excluding patients who had iritis for less than 6 months post-operatively, history of diabetes became significant, in addition to African American race. Table 1 summarizes the patient demographic and pre-operative/intra-operative characteristics.

**Table 1:** Comparison of characteristics between patients with persistent post-operative iritis versus control group with associated *p* values

Characteristic	Control group	Iritis group					
Sample size (*n*)	All control patients	All iritis patients	*p* value	Excluding prior inflammation/diagnosis	*p* value	Iritis > 6 months and no prior Inflammation/diagnosis	*p* value
Patients	40	39	–	22	–	9	–
Demographic							
Mean age (years) ± standard deviation	66.7 ± 10.4	65.8 ± 14.1	0.76	69.1 ± 12.5	0.417	70.8 ± 16.6	0.345
Female sex [*n* (%)]	25 (63)	30 (77)	0.163	17 (77)	0.234	6 (67)	1.00
African American race [*n* (%)]	11 (28)	34 (87)*	< 0.001	22 (100)*	< 0.001	9 (100)*	< 0.001
Clinical history [*n* (%)]							
Prior ocular inflammation	0	13 (33)		–	–	–	–
Prior ocular trauma	0	2 (5)	0.241	1 (5)	0.355	1 (11)	0.184
Comorbidities [*n* (%)]							
Underlying systemic diagnosis†	0	9 (23)		–	–	–	–
Glaucoma	13 (33)	11 (28)	0.678	5 (23)	0.417	3 (33)	1.00
Diabetes	11 (28)	12 (31)‡	0.749	11 (50)	0.076	6 (67)*	0.049
Intra-operative finding [*n* (%)]							
Use of ring/hooks	1 (3)	9 (23)*	0.007	2 (9)	0.285	2 (22)	0.083

*Significant at the 0.05 probability level

†The diagnoses were as follows: ulcerative colitis (1), sarcoidosis (4), multiple sclerosis (2), rheumatoid arthritis (1), ankylosing spondylitis (1), and HSV (1)

‡Of the 12 patients in this group, 0 had a recent intravitreal injection, and the peri-operative HbA_1c_ ranged from 5.9–7.8%. Four of these patients had documented DR without cystoid macular edema (CME). One patient with diabetes was noted to have CME not due to DR. Two other patients were found to have pre-existing CME without the diagnosis of diabetes

The analysis of uveitis patients showed that 13 patients had a history of prior ocular inflammation. The types of uveitis cases reported were as follows: panuveitis (1); iritis (4); anterior and intermediate uveitis (2); episcleritis (2); and uveitis, not further specified (5). Four patients were taking prednisone or a disease-modifying antirheumatic drug at baseline. Four of the cataracts were described as uveitic. Intra-operatively, pupil expansion devices were used in 6 cases (5 iris hooks and 1 malyugin ring). No cases were complicated by a posterior capsular tear or anterior vitrectomy. The average duration of iritis amongst these patients was 5.8 (± 5.0) months, and there were 7 cases of iritis lasting greater than 6 months. Post-operatively, about 50% of patients reported iritis symptoms (6 patients/8 cases) including pain, photophobia, and/or blurry vision. Additionally, 50% showed a rapid improvement with additional treatment.

Table 2 provides a detailed summary of findings amongst diabetic patients in the iritis and control groups.

**Table 2:** Comparison of diabetic history and demographics between the iritis and control groups

	Iritis patients	Control patients
Diabetic patients	12	11 (16 cases)
+ Diabetic retinopathy	4	3
+ Diabetic macular edema	1	0
+ Recent intravitreal injection	0	0
Female sex	9	6
African American race	11	6
Average HbA_1c_	6.42% (5/12 known)	6.83% (10/16 known)
Average duration of diabetes	49 months (3/12 known)	50.7 months (7/16 known)

There were 19 patients diagnosed with glaucoma or as a glaucoma suspect receiving treatment. Table 3 summarizes the treatment differences between the iritis and control groups. There were no statistical differences between the two groups.

**Table 3:** Treatment comparison of glaucoma or glaucoma suspect patients

Treatment	All patients	Iritis patients	Control patients
Medical			
Alpha agonist	6	3	3
Carbonic anhydrase inhibitor (topical)	8	4	4
Beta blocker	11	5	6
Prostaglandin analogue	10	5	5
Direct cholinergic agonist	1	0	1
Carbonic anhydrase inhibitor (oral)	1	1	0
Laser			
Selective laser trabeculoplasty	5	2	3
Laser peripheral iridotomy	3	2	1

**Fig. 2:**
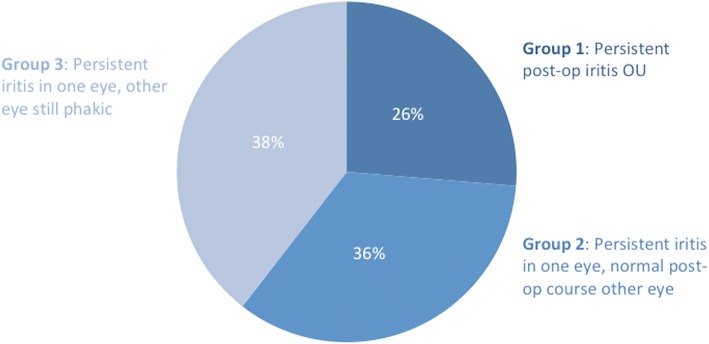
Percentage of patients by group. Legend: Patients in the prolonged post-iritis group were divided into three groups based on the course of the other eye and if both eyes had undergone surgery

The patients with prolonged iritis were separated into groups according to whether one or both eyes were affected (see Fig. 2). Group 1 (10 patients) had persistent post-operative iritis in both eyes. Group 2 (14 patients) had both eyes operated, but only one eye had persistent iritis, which was evenly divided between the first or second eye being affected. Group 3 (15 patients) had persistent iritis in the one eye, but were still phakic in the other eye. All of the patients in group 1 were females, whereas the majority of patients in group 2 were males. Group 2 was compared to group 1 to assess the characteristics of cases where both eyes underwent cataract surgery and the one eye versus both eyes was affected. In group 2, 2 patients had iritis only in the eye where a pupil expansion device was used compared to the fellow eye where it was not used. Similarly, the patient with prior ocular trauma had prolonged iritis only in the affected eye. This analysis suggests that these factors may have a correlation with persistent iritis in this subset of patients, but the sample size is too small to derive statistical significance. History of diabetes was not statistically significant between groups 1 and 2.

Assessment of type and grade of the cataracts revealed that all patients in the control group had senile cataracts, whereas 4 patients in the persistent iritis group had uveitic cataracts (the remainder were senile). Twenty-nine of the 66 eyes (43.9%) in the control group had grade 3 or greater cataracts versus 15 of the 49 eyes (30.6%) in the persistent iritis group.

Intra-operative characteristics of both groups were analyzed. Femtosecond laser-assisted surgery was used for 23 cases in the control group and 2 cases in the iritis group. Table 4 summarizes the type of pupil expansion device used. One patient in the iritis group had IFIS requiring anterior vitrectomy (no cases of IFIS in the control group). That was also the only case where a sulcus IOL was placed (versus posterior chamber IOL in all other patients in both groups). Nine of the surgeries in the control group were combined with other procedures (e.g., trabeculectomy) versus only 3 of the surgeries in patients who developed persistent iritis. In the persistent iritis group, intracameral antibiotics were given in 85.7% of cases in the persistent iritis group and 95.4% of cases in the control group.

**Table 4:** Pupil expansion devices used for cases in the iritis and control groups and comparison of device choice based on inflammation history

	Malyugin ring	Iris hooks
Total number of cases	5	5
Iritis group	4	5
Control group	1	0
Cases with prior ocular inflammation	1	5
Cases with known inflammatory disease	1*	3†

*Ulcerative colitis and ankylosing spondylitis (1)

†Multiple sclerosis (2) and sarcoidosis (1)

Post-operatively, average BCVA in the control group was 0.16 (± 0.17) logMAR, and the average BCVA in the persistent iritis group was 0.26 (± 0.43) logMAR. Average maximum IOP was 20 in the control group and 16 in the iritis group. Further analysis of the persistent iritis group is summarized in Table 5. Compliance ratings were assigned based on attendance at scheduled follow-up visits and attending comments on adherence to treatment regimens. Two patients (2 eyes) were noted to have poor compliance.

**Table 5:** Post-operative symptomatology, exam findings, and treatment course in the prolonged iritis group

	Percentage of eyes	Number of eyes
Moderate to severe inflammation*	59	29
Active symptoms†	82	40
Indolent, persistent course without rapid response to treatment	35	17
Supplemental therapy with oral prednisone	8	4

*Defined as having at least 2+ cell or moderate/3+ flare

†The most common symptom expressed was pain (26 eyes), followed by photosensitivity (15 eyes), and blurry or decreased vision (11 eyes)

## Discussion

This is the first report of risk factors associated with prolonged post-operative iritis in patients who underwent uncomplicated cataract surgery by phacoemulsification and who did not have a history of uveitis or other underlying inflammatory disorders. Prolonged post-operative iritis represents a significant burden amongst uveitis cases seen in the southeastern part of the USA. A study of uveitis diagnoses at the University of Virginia over a 30-year period revealed that 10% of all cases of uveitis were “post-procedural,” with 48.7% of those cases (approximately 5% of the total burden) associated with cataract extraction and IOL implantation [6]. In our study, there was a significant association between a diagnosis of diabetes and an extended duration of post-operative iritis lasting longer than 6 months. African American race was also significantly correlated with prolonged post-operative iritis when predisposing factors such as known prior uveitis episode(s) or an underlying inflammatory disease diagnosis were excluded.

The association of diabetes with prolonged post-operative inflammation after cataract surgery is perhaps not surprising in light of the link between inflammation and diabetes [7]. In recent years, the theory that obesity leads to chronic systemic inflammation by activation of immune cells with the release of inflammatory mediators, and thereby increased the risk of metabolic disorders such as diabetes, has become well established [8]. Several studies have confirmed higher levels of flare correlating with inflammatory markers in the aqueous fluid in patients who are diabetic compared to non-diabetic patients [9, 10]. Higher levels of flare are associated with a breakdown of the blood-aqueous barrier in diabetic patients and may persist for many months after cataract surgery [11]. It may be theorized that persistent hyperglycemia and a chronic systemic pro-inflammatory state predispose diabetic patients to a prolonged course of post-operative iritis refractory to standard 1-month topical steroid treatment tapers.

There have been several studies that have examined the differences in aqueous markers in diabetic and non-diabetic patients after cataract surgery. Miric et al. studied lens oxidative stress markers in patients after senile cataract extraction. Serum and lens levels of xanthine oxidase were higher in the diabetic than the non-diabetic group, and they positively correlated with HbA_1c_ concentration, suggesting that the diabetic state could possibly contribute to earlier cataract formation [12]. Additionally, Mitrovic et al. found significantly altered levels of inflammatory mediators in the aqueous humor of diabetic patients following cataract surgery, leading to a predisposition for corneal edema [13]. While the exact mechanism behind increased inflammation in diabetic eyes is unknown, there are probably many potential pathways involved. Prolonged inflammation within the eye may not follow the same patterns as inflammation elsewhere in the body due to its immune-privileged state.

African American race remained a statistically significant factor in all comparisons. This may be attributed to the fact that the African American race is an independent risk factor for inflammation and oxidative stress, positing that the inflammatory response following cataract surgery may be more robust in these patients [14].

Unsurprisingly, intra-operative pupil expansion device use correlated with prolonged post-operative iritis, likely due to iris trauma during insertion and manipulation of pupil expansion devices leading to disruption of the blood-aqueous barrier. The density of the cataract or history of previous ocular trauma did not appear to have an increased risk of prolonged post-operative inflammation in this study.

The incidence of persistent iritis in our study was 0.32%, which is similar to that previously reported by Patel et al. [3]. The incidence was 0.24% in Patel’s study with the exclusion criteria similar to those of our own study: duration of iritis less than 6 months, prior uveitis or underlying systemic disease, penetrating trauma, endophthalmitis, retained lens material, neovascular glaucoma, and prior intraocular surgery.

This study is limited by sample size as well as the expected limitations of any retrospective data collection. The results were confined to information available in the electronic medical record and were subject to variability in provider documentation (e.g., various exam documentation styles, grading scales, codes), which can introduce bias. The incidence rate may be underestimated because the charts for the CPT data query were not all reviewed, so some cases of prolonged post-operative inflammation may have been missed. Certain parameters such as patient symptomatology and compliance with treatment regimen could not be evaluated unless they were deliberately reported in the charts. Multivariate analyses could not be run due to the relatively small sample size, which was partially limited by the ability to perform a comprehensive chart review since some information did not transfer or was not easily searchable during or pre-dating the conversion to electronic medical records. Despite these limitations, this report provides an extremely important first step in the evaluation of risk factors for prolonged post-operative iritis, which have not been previously reported in the literature.

Overall, in both groups, the post-operative refractive outcomes were excellent. Long-term refractive outcomes after cataract surgery are dependent on many factors, including pre-existing ocular morbidity, intra-operative complications, and development of post-operative sequelae such as cystoid macular edema or retinal detachment. While a prolonged post-operative inflammatory course may not necessarily lead to a worse visual acuity outcome, it can be associated with delayed post-operative visual recovery and with patient discomfort. Supplementary peri-operative medications can improve control of post-operative outcomes in patients who are at high risk pre-operatively for having prolonged or rebound post-op iritis.

## Conclusions

This analysis showed that diabetes was a significant independent risk factor for the development of chronic iritis lasting greater than 6 months in patients without a history of inflammation. Additionally, African American race was significantly correlated with post-operative iritis lasting greater than 1 month. We propose that altering the peri-operative treatment regimen for patients who are diabetic and/or African American could shorten and improve their post-operative recovery. Further investigation with a large-scale prospective trial would allow for a more powerful statistical analysis and may help to explain how these factors may contribute to post-operative inflammation.

## Abbreviations

BCVA: Best-corrected visual acuity
DR: Diabetic retinopathy
FLACS: Femtosecond laser-assisted cataract surgery
HbA_1c_: Glycated hemoglobin; hemoglobin A_1c_
ICD: International Classification of Diseases
IFIS: Intra-operative floppy iris syndrome
IOL: Intraocular lens
IOP: Intraocular pressure
MIGS: Minimally invasive glaucoma surgery
MUSC: Medical University of South Carolina
RF: Rheumatoid factor
SPARC: Services, Pricing, and Application for Research Centers
